# Local Transformations of Androgens into Estradiol by Aromatase P450 Is Involved in the Regulation of Prolactin and the Proliferation of Pituitary Prolactin-Positive Cells

**DOI:** 10.1371/journal.pone.0101403

**Published:** 2014-06-30

**Authors:** María José García Barrado, Enrique J. Blanco, Marta Carretero Hernández, María Carmen Iglesias Osma, Manuel Carretero, Julio J. Herrero, Deborah Jane Burks, José Carretero

**Affiliations:** 1 Department of Human Anatomy and Histology, Faculty of Medicine, University of Salamanca, Spain; 2 Laboratory of Neuroendocrinology, Institute of Neurosciences of Castilla y León, and Laboratory of Endocrine-Metabolic Diseases of IBSAL, University of Salamanca, Spain; 3 Department of Physiology and Pharmacology, Faculty of Medicine, University of Salamanca, Spain; 4 Faculty of Human and Social Sciences, University Pontificia of Salamanca, Spain; 5 Department of Surgery, Faculty of Medicine, University of Salamanca, Spain; 6 Laboratory of Neuroendocrinology, Centro de Investigación Príncipe Felipe (CIPF), Valencia, Spain; University of Cordoba, Spain

## Abstract

In previous studies we demonstrated the immunohistochemical expression of aromatase in pituitary cells. In order to determine whether pituitary aromatase is involved in the paracrine regulation of prolactin-producing pituitary cells and the physiological relevance of pituitary aromatase in the control of these cells, an *in vivo* and *in vitro* immunocytochemical and morphometric study of prolactin-positive pituitary cells was carried out on the pituitary glands of adult male rats treated with the aromatase antagonist fadrozole. Moreover, we analyzed the expression of mRNA for the enzyme in pituitary cells of male adult rats by *in situ* hybridization. The aromatase-mRNA was seen to be located in the cytoplasm of 41% of pituitary cells and was well correlated with the immunocytochemical staining. After *in vivo* treatment with fadrozole, the size (cellular and nuclear areas) of prolactin cells, as well as the percentage of prolactin-positive cells and the percentage of proliferating-prolactin cells, was significantly decreased. Moreover, fadrozole decreased serum prolactin levels. *In vitro*, treatment with fadrozole plus testosterone induced similar effects on prolactin-positive cells, inhibiting their cellular proliferation. Our results suggest that under physiological conditions aromatase P450 exerts a relevant control over male pituitary prolactin-cells, probably transforming testosterone to estradiol in the pituitary gland.

## Introduction

Of the two pathways through which androgens are metabolized -reduction and aromatization- the latter depends on the presence of an enzyme (aromatase P450) belonging to the family of cytochrome P450.

There is growing awareness that androgens and estrogens play general metabolic roles that are not directly involved in reproductive processes. Estrogen is no longer considered solely an endocrine factor, but instead is produced in a number of extragonadal sites and acts locally at these sites in a paracrine and autocrine fashion. These sites include breast, bone, vasculature, and brain. Within these sites, the action of aromatase can generate high levels of estradiol locally, without significantly affecting circulating levels [1], [2].

At our laboratory, we have demonstrated the expression of aromatase in the rat pituitary gland. This expression was related to the sex and age of the animals. Moreover, we reported a very strong correlation between the pituitary expression of aromatase and the incidence of spontaneous prolactinomas in old rats [3] and humans [4]. Similar results were later reported by different authors in non-tumoral pituitaries of humans and different animal species [5]–[11] and in human prolactinomas [12]. However, the physiological relevance of aromatase and the possible aromatization of testosterone to estradiol in the regulation of pituitary hormones are not well known.

Because estradiol is an important physiological regulator of pituitary prolactin secretion [13]–[19] and is involved in the control and proliferation of prolactin-producing cells [20], the aim of the present study was to analyze the relevance of the regulatory role of the local transformation of androgens into estradiol by aromatase in the maintenance of the population of prolactin cells in the male pituitary gland, through the pituitary aromatization of testosterone to estradiol. For this purpose, the *in vivo* or *in vitro* effects of aromatase on the pituitary glands of adult male rats were blocked by treatment with the aromatase antagonist fadrozole.

## Materials and Methods

### Animals

Animal experimentation was performed according to the Guide for the Care and Use of Laboratory Animals of the National Institutes of Health (NIH Publication No. 85–23, revised 1996). All procedures were approved by the Committee for the Care and Use of Animals of the University of Salamanca, which ensures compliance with national and European legislation regarding the use of animals in research (Spanish RD 53/2013 and 2010/63/EU).

Fifty Wistar male adult rats (175–200 g body weight) were used. The animals were divided into 5 groups of 10 animals each according to treatment: (a) untreated animals; (b) control 1-dose animals, treated intramuscularly with 1 dose of 200 µl of saline; (c) control 5-dose animals, treated intramuscularly with 5 doses (1 dose per day) of 200 µl of saline; (d) 1-dose-treated animals, treated intramuscularly with 1 dose of 0.5 mg of fadrozole in 200 µl of saline; (e) 5-dose-treated animals, treated intramuscularly with 5 doses (1 dose per day) of 0.5 mg of fadrozole in 200 µl of saline.

During the experiments, all groups were kept under standard stabling conditions (temperature 21±2°C, relative humidity 50±5%, controlled photoperiod of 14 h light/10 h darkness, food and water *ad libitum* with a balanced rat/mouse maintenance diet (Panlab).

### Sample collection and processing

Animals were sacrificed between 10.00 and 11.00 h by decapitation after anaesthesia by isofluorane inhalation. The pituitary glands were carefully dissected out and immediately fixed in a solution of 15% saturated picric acid in 4% paraformaldehyde in 0.1 M phosphate buffer, pH 7.4, for 24 h. Then, they were dehydrated in ethanol, cleared with xylene, and embedded in paraffin in order to obtain coronal serial sections of 5 µm thickness. These were placed on slides treated with gelatin-chrome alum and were then used for the immunohistochemical study. After sacrifice, blood samples were obtained to determine serum prolactin levels.

### Pituitary cultures

Following anaesthesia with isoflurane, 5 male Wistar rats (175–200 g) were killed by decapitation and the anterior pituitary glands were removed and washed in Earle’s balanced salt solution. Enzymatic dispersion was carried out by incubation for 15 minutes at 37°C in Hank’s solution to which 0.15% MgCl_2_, 0.1% papain, 0.01% DNase and 0.1% of neutral protease had been added. Mechanical dispersion was achieved by passing the pituitaries through Pasteur pipettes and 20 to 22 gauge needles. After centrifugation, the supernatant was removed and the cells were suspended in an appropriate volume of Dulbecco’s modified Eagle’s medium (DMEM), supplemented with 10% calf serum, 2.5% foetal calf serum, 2% L-glutamine, 1000 IU/ml of penicillin and 1000 IU/ml of streptomycin. The cells were seeded on 60 culture chamber slides (Nunc, 1 ml) at a final concentration of 2.5×10^5^ cells/ml and incubated at 37°C in a 5% CO_2_/95% air atmosphere for 7 days. On the 4th day of incubation the medium was changed by fresh medium. On the 7th day, cultures were treated with 10^−6^ M testosterone, 10^−6^ M Fadrozole and incubated for 1, 3, 6 or 12 hours (five chambers per treatment and time-point assayed). Previously, testosterone was diluted in ethanol, the final concentration of ethanol in the treated and control dishes being 0.0027%.

After treatment, the chambers were carefully washed with Dubelcco’s sterile PBS and then the cells were fixed in Somogy’s solution for 1 hour, followed by careful rinsing in PBS. In order to validate the results obtained, two pituitary cultures were made under identical experimental conditions.

### Serum prolactin determination

From serum samples, prolactin was assayed by double antibody RIA using the Dipesarat hormone kit according to the manufacturer’s instructions. The intra- and inter-assay coefficients of variation were lesser than 3% and 6% respectively.

### Western blotting

**Figure 1:**
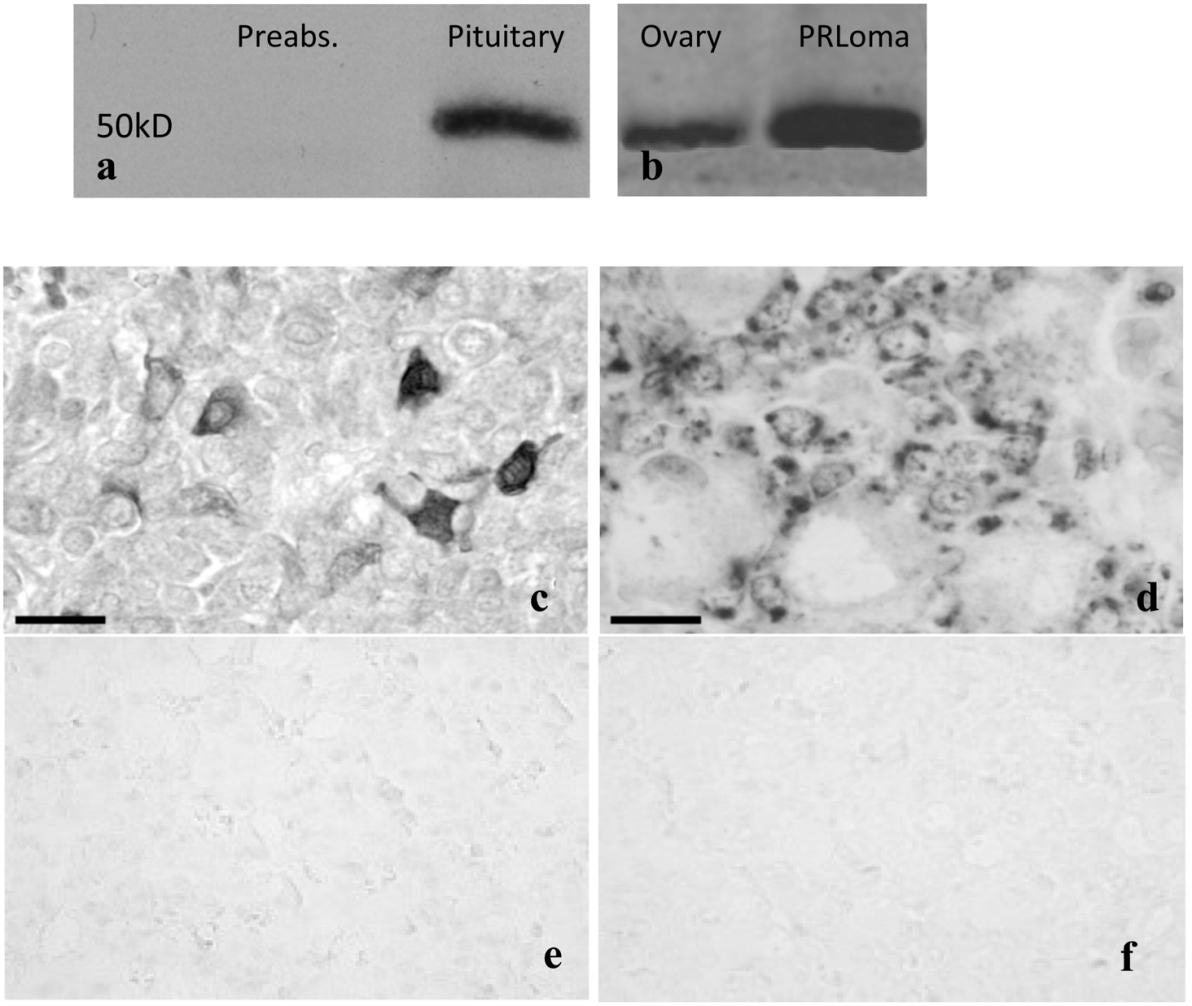
Expression of aromatase by Western blot. a) preabsorption and expression in the pituitary, b) positive controls from the ovary of a female adult rat, and from a prolactinoma from a female old rat. Images showing the expression of aromatase in the pituitary of adult male rats. c) Aromatase-positive cells revealed by immunohistochemistry. d) mRNA-aromatase-positive cells detected by in situ hybridization. Negative controls for in situ hybridization: e) absence of the probe and f) sense aromatase probe. Scale bar: c, d: 50 µm.

For Western blotting, the pituitary glands were dissected from adult rats and immediately frozen. Tissues were then disrupted by homogenization in lysis buffer (137 mM NaCl, 10 mM TB, pH 7.4, 10% glycerol and 1% Triton X-100, containing a cocktail of protease inhibitors). Insoluble material was removed from the lysates by centrifugation at 10,000 rpm for 10 min. Protein concentrations were determined using the standard Bradford assay. Fifty µg of total protein from each rat sample was separated by 10% SDS-PAGE. Following electrophoresis, proteins were transferred to nitrocellulose and then blocked for 1 h with 5% non-fat dry milk in PBS. The nitrocellulose membranes were then incubated overnight with either preabsorbed anti-aromatase serum or anti rat aromatase P450 rabbit polyclonal serum (Rb-SG 872 Sigma), diluted 1∶500. Blots were subjected to 3×15 min washes with PBS and then incubated for 1 h with HRP-labelled secondary antibodies (1∶10,000 in PBS). Following extensive washing, the blots were revealed by Amersham ECL western blotting detection reagents (Amersham). The average exposure time was 2 min. Western blotting revealed the existence of a protein with a molecular weight of around 50 kDa in the pituitary gland (Figure 1a).

For preabsorption tests, in western blotting or immunohistochemistry diluted anti-aromatase serum (1∶500) was preabsorbed (24 h at 4°C) with the peptide sequence C-EIIFRHIFNTPFLQC [21], corresponding to residues 489–503 of rat aromatase (50 µg peptide/ml antibody solution), which was used to obtain rabbit polyclonal anti rat aromatase P450 rabbit polyclonal serum (Rb-SG 872 Sigma). Positive controls from ovary and prolactinoma (Figure 1b) were included.

### Immunohistochemistry

Immunohistochemical studies were performed using the streptavidin-peroxidase method in single immunostaining, or the strepto-avidin-peroxidase and PAP methods in double immunostaining. The sources and working dilutions of the primary anti-sera were as follows: anti rat aromatase P450 rabbit polyclonal serum (Rb-SG 872 Sigma, diluted 1∶500), anti-Proliferating Cell Nuclear Antigen (PCNA) monoclonal antibody (Dako, diluted 1∶3000) and anti-prolactin polyclonal antibody (Dako, diluted 1∶1000).

Single immunostaining was carried out for aromatase and prolactin: Primary antibodies were incubated with tissue sections at 4°C overnight. After washing, the slides were incubated for 45 min at room temperature with biotinylated-goat anti-rabbit or mouse IgG (Caltag, diluted at 1∶100 in TBS) and then for 45 min at room temperature with streptavidin-peroxidase complex (Caltag, diluted at 1∶150). The reaction was developed with freshly prepared 3-3′-diaminobenzidine (Sigma, 0.024% in TB buffer plus 0.03% H_2_O_2_). The washes and antibody dilutions were made in TBS: HCl-Trizma, 0.05 M, pH 7.4, plus 0.8% NaCl.

To study PCNA-positive cells and to determine the PCNA-Prolactin labelling index, a double labelling immunohistochemical method for PCNA and prolactin was developed. Endogenous peroxidase was blocked with H_2_O_2_ in methanol and non-specific reactions of the secondary antibody by incubation in normal goat serum (Dako, diluted 1∶30). Sections were incubated overnight at 4°C with mouse anti-PCNA monoclonal antibody (PC10, Dako, diluted 1∶3000 in TBS). Biotinylated goat anti-mouse IgG (Dako, diluted 1∶100) and Avidin-Biotinylated horseradish peroxidase complex (ABC kit, Dako, diluted 1∶100) were successively applied at room temperature for 40 min and 30 min, respectively. The reaction was developed in freshly prepared 3,3′-DAB (0.025% in TB buffer containing 0.03% of H_2_O_2_). Following PCNA immunolabelling, the peroxidase-antiperoxidase (PAP) reaction was performed for the detection of prolactin, The reaction was allowed to progress by incubation for 40 min at room temperature with swine anti-rabbit IgG (Dako, diluted 1∶100), and later with incubation with soluble peroxidase anti-peroxidase complex (Dako diluted 1∶100) for 35 min at room temperature. The reaction was developed in freshly prepared 4-chloro-1-naphthol (1.7×10^−3^ M in 3% absolute ethanol and TB-buffer containing 0.3% H_2_O_2_).

Preabsorption tests with prolactin and tests replacing the specific serum by normal non-immune rabbit serum abolished the reaction. Using ELISA, the specificity of swine anti-rabbit IgG was lower than 1% for rat and mouse IgG and 100% for rabbit IgG. For the washes and dilutions of the sera, TB buffer (0.05 M, pH7.4) containing 0.8% NaCl was used. The reaction was developed in freshly prepared 4-chloro-1-naphthol (1.7×10^−3^ M in 3% absolute ethanol and TB-buffer containing 0.3% H_2_O_2_).

### In situ hybridization

These studies were performed using a non-isotopic method involving the immunocytochemical detection of biotin using the streptavidin-biotin-peroxidase method. To accomplish in situ hybridization, the sense biotinylated oligonucleotide 5′BIO-gag gat gac gtg att gac ggc tac ccg gtt aaa aag gga act aac atc att ctg aac atc gga, and antisense 5′ Bio-ctc cta ctg cac taa ctg ccg atg ggc caa ttt ttc cct tga ttg tag taa gac ttg tag cca, 100% specific to rat aromatase P450 according to the GenBank data base (accession number, M33986), was used as probes [21].

Slides were prehybridized in Omnibuffer for 30 minutes at 37°C. Hybridization with the biotinylated-probe (100 pg/ml in Omnibuffer) was carried out using a Hybaid thermocycler overnight at 37°C. The reaction was stopped by washes in 1 x SSC at 54°C for 20 min, 1 x SSC at room temperature for 20 minutes, and for 20 minutes in PBS (0.01 M, pH 7.4, plus 0.8% NaCl). Biotin was detected using monoclonal anti-biotin antibodies (Roche, 1∶250 in TBS: 0.05 M HCl-Trizma, pH 7.4, plus 0.8% NaCl) overnight at 4°C in a humidity chamber, followed by biotinylated goat anti-mouse (Caltag, 1∶250 in TBS). The reaction was amplified using the tyramide amplification kit (Dako) according to the instructions of the manufacturers. The final reaction was developed with 3,3′-diaminobenzidine (0.025 M, Sigma, in 0.05 M TB-HCl buffer, pH 7.4) to which 0.03% H_2_O_2_ had been added. The slides were counterstained using Mayer’s acid haematoxylin.

As controls, hybridization with sense probe, omission of the probe and pretreatment with RNase were performed, no reaction being observed in any case (Figures 1e and 1f).

### Quantification of aromatase mRNA-expressing cells

The percentage of aromatase mRNA-expressing cells was quantified in each animal following the double-blind procedure. Briefly, eight thousand cells (with intact cellular and nuclear profiles) were counted from 20 sections, separated from one another by at least 50 µm (400 cells/section), chosen randomly from all parts of the gland, after which the percentage of reactive cells was calculated.

### Quantification of immunoreactive cells

Immunopositive cells were quantified following the double-blind procedure. Briefly, *in vivo* four thousand cells (with intact cellular and nuclear profiles) were counted from 20 sections separated from one another by at least 50 µm (200 cells/section), chosen randomly from all parts of the gland, after which the percentage of aromatase-, prolactin- or PCNA and prolactin-positive cells was calculated. *In vitro,* 400 cells chosen randomly from all parts of the dish were counted, and the percentage of prolactin-positive cells with respect to the total number of cells, and the percentage of PCNA- and prolactin-positive cells, of total prolactin-positive cells, was calculated.

### Morphometry

Using the Image J software (NIH), 500 randomly chosen prolactin-positive cells per animal, or 100 randomly chosen prolactin-positive cells per dish were measured for morphometric study (cells were considered when the nuclear and cellular profiles in the plane of section or focus were clearly distinguished). Cellular and nuclear areas were determined.

### Statistical analyses

For each parameter evaluated, the values obtained, after each treatment, were processed statistically using the SPSS software and the differences observed among mean values were compared using analysis of variance, accepting p values of <0.05 as significant for the Scheffé F test. Results are expressed as arithmetic means ± standard error of the mean.

## Results

### Aromatase P450 expression in the pituitary gland of male adult rats

#### Western blotting

The image in Figure 1 shows that the hypophysis of the adult male rat contains a protein of 52 kDa, which is detected with the Rb-872 antibody, specific for rat aromatase P450. The immunoblot did not appear after preabsorption (Figure 1a).

#### Inmunohistochemistry

As shown in Figure 1c, in many hypophyseal glandular cells of adult male rats the presence of the enzyme was detected by means of immunohistochemistry.

#### In situ hybridization

Figure 1d shows that in many hypophyseal cells of adult male rats it is possible to detect the presence of aromatase mRNA by *in situ* hybridization (controls: absence of the probe –Fig. 1e, and sense aromatase Fig. 1f).

### In vivo effects of fadrozole

#### Prolactin levels

**Figure 2:**
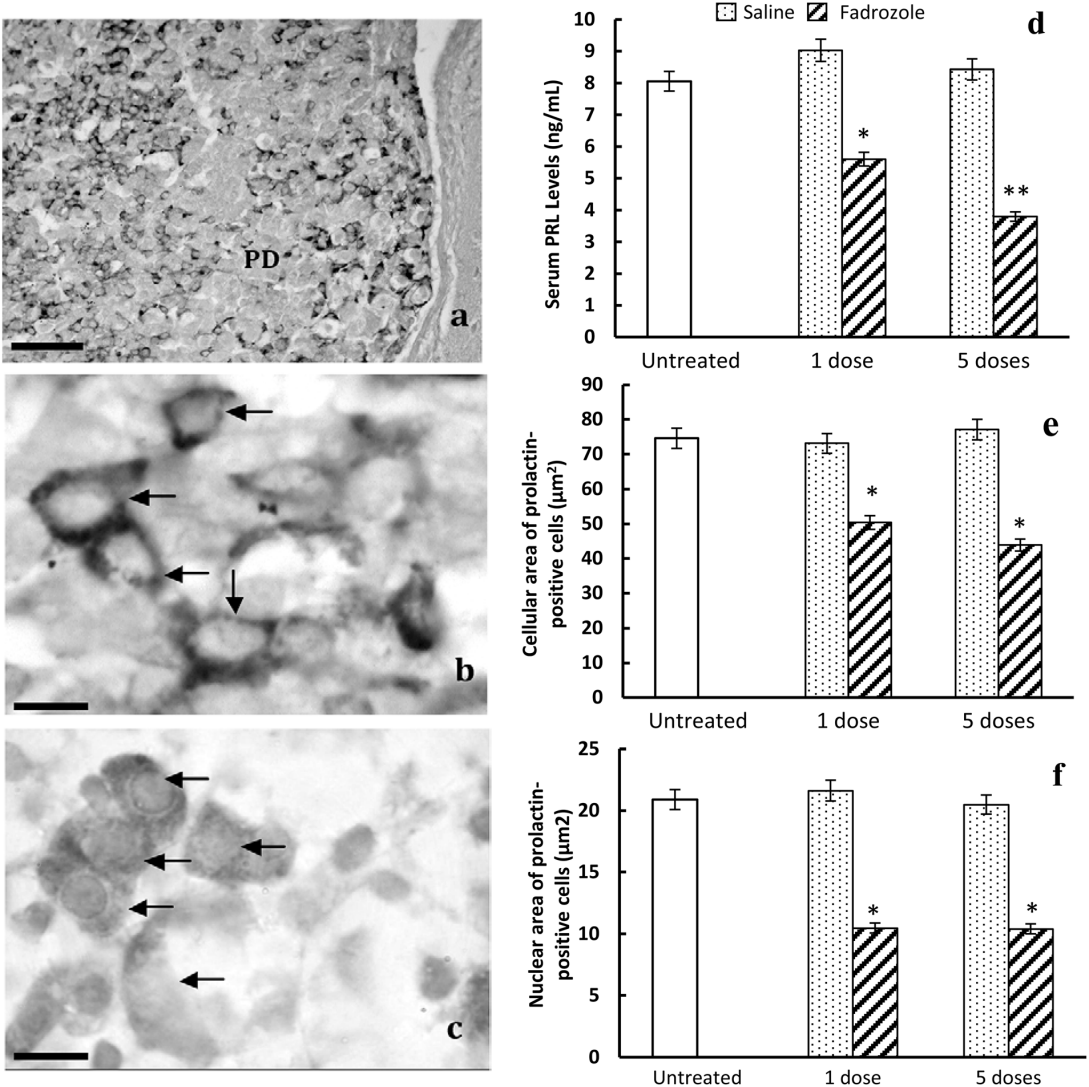
Effects of fadrozole on prolactin-positive cells in the pituitary of male rats. a) General disposition of prolactin-positive cells in the gland. PD: pars distalis of pituitary gland. Scale bar: 100 µm. b) Irregular and strongly stained prolactin-positive cells (arrows) in untreated male rats. c) Polygonal and weakly stained prolactin-positive cells (arrows) after 5 doses of fadrozole. Scale bar b, c: 12 µm d) Plot showing the *in vivo* effects of the 1 or 5 doses of fadrozole on the serum levels of prolactin (ng/mL) (*p<0.01 with respect to untreated and control animals, **p<0.01, with respect to untreated and control animals and p<0.05 with respect to 1 dose of fadrozole-treated animals). Morphometric effect of treatment with fadrozole on cellular (e) or nuclear (f) areas (µm^2^); in both cases fadrozole induces decreases in size (*p<0.01 with respect to untreated and control animals).

The untreated and control (saline-treated) animals showed similar basal prolactin serum levels (see Figure 2d). Treatment with fadrozole led to a clear and significant (p<0.01) decrease in serum prolactin levels, and significant differences were found among animals treated with one dose of fadrozole and those treated with 5 doses (p<0.05).

#### Morphological findings in prolactin-positive cells

The prolactin cells of the untreated animals were found scattered throughout the pituitary gland, with a predominance of polygonal and oval shapes, with scant but very reactive cytoplasm and a central nucleus. There were also cells with a polarized nucleus. (Figure 2a and arrows in Figure 2b). In both groups of treated animals (1 and 5 doses of fadrozole) the intensity of the cytoplasmic reaction of prolactin-positive cells was lower than in the untreated or control animals (arrows in Figure 2c).

#### Morphometric findings in prolactin-positive cells

The mean cellular area of the prolactin-positive cells (Figure 2e) of the untreated animals was 74.58±1.65 µm^2^. Non-significant variations were observed after treatment with saline (1 dose: 73.11±1.99 µm^2^, and 5 doses: 77.08±1.35 µm^2^). Treatment with fadrozole decreased the cellular area of prolactin-positive cells significantly (1 dose: 50.4±1.35 µm^2^; 5 doses: 43.89±1.36 µm^2^ p<0.01 with respect to untreated and saline-treated animals). Very similar results were found when mean nuclear areas (Figure 2f) were analysed, with similar values for untreated animals (20.91±0.51 µm^2^) and saline-treated animals (1 dose: 21.62±0.24 µm^2^; 5 doses: 20.48±0.38 µm^2^) and significant decreases (p<0.01 with respect to untreated and saline-treated animals) following fadrozole treatment (1 dose: 10.48±0.26 µm^2^; 5 doses: 10.41±0.18 µm^2^).

#### Percentage of prolactin-positive cells

**Figure 3:**
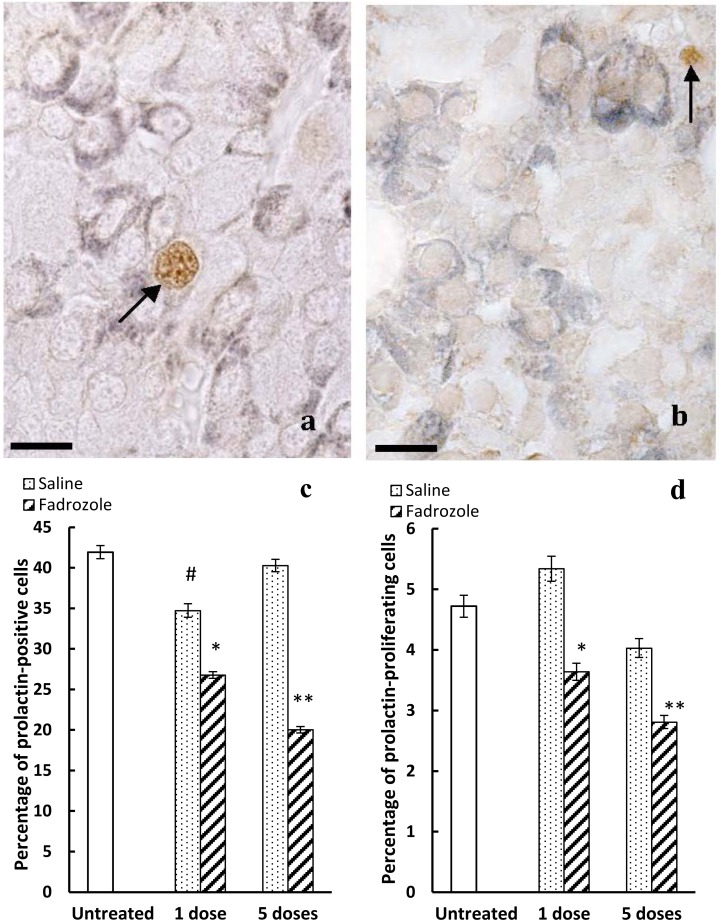
Effects of *in vivo* treatment with fadrozole on the cellular proliferation of prolactin-positive cells. a) Double labelled immunohistochemistry for prolactin (dark blue-grey) and PCNA (brown). In untreated animals, few prolactin-positive cells are labelled for PCNA (arrow). Scale bar: 12 µm. b) After 5 doses of fadrozole it is uncommon to find cells labelled jointly for PCNA and prolactin (arrow points to PCNA- but not prolactin-positive cells). Scale bar: 12 µm. c) The percentage of prolactin-positive cells decreases significantly with respect to the untreated or control animals after 1 dose (**p<0.01 with respect to untreated animals, and p<0.05 with respect to control animals) or 5 doses (**p<0.01 with respect to untreated and control animals and p<0.05 with respect to 1 dose of the fadrozole treated animals). After 1 dose of saline the percentage decreases with respect to untreated animals (#p<0.05), but not after 5 doses. d) Fadrozole decreases the proliferation of prolactin-positive cells (*p<0.05 with respect to untreated and control animals; **p<0.01 with respect to untreated and control animals and p<0.05 with respect to 1 dose of fadrozole-treated animals).

The percentage of prolactin-positive cells (Figure 3c) in untreated animals was 41.9±0.8%. This percentage decreased after saline treatment. However, this decrease was only significant after 1 dose of saline (34.7±0.7, p<0.05 with respect to untreated animals) but not after 5 doses (40.3±0.8). Treatment with fadrozole significantly decreased the percentages of prolactin-positive cells alter 1 dose (26.8±0.5, p<0.01 with respect to untreated and saline-treated animals) and this effect was more evident after 5 doses (20.0±0.5, p<0.01 with respect to untreated and saline-treated animals, and p<0.05 with respect to fadrozole 1-dose treated animals).

#### Percentage of prolactin-proliferating cells

Prolactin-proliferating cells appeared with brown-stained nuclei and a dark blue-stained cytoplasm (Figures 3a, 3b). The percentage of prolactin-proliferating cells (Figure 3d) was relatively low in untreated males (4.7±0.2) and very similar values were found for the saline 1-dose control animals: 5.3±0.2 and saline 5-dose rats: 4.0±0.1. Treatment with fadrozole significantly decreased the percentages of prolactin-proliferating cells. This effect was more relevant in the animals treated with 5 doses of fadrozole (2.8±0.1, p<0.01 with respect to untreated and saline 5 doses animals) than in the animals treated with 1 dose of the aromatase antagonist (3.6±0.1, p<0.05 with respect to untreated, saline 1-dose and fadrozole 5-dose animals).

### In vitro effects of fadrozole

#### Morphometric findings in prolactin-positive cells

**Figure 4:**
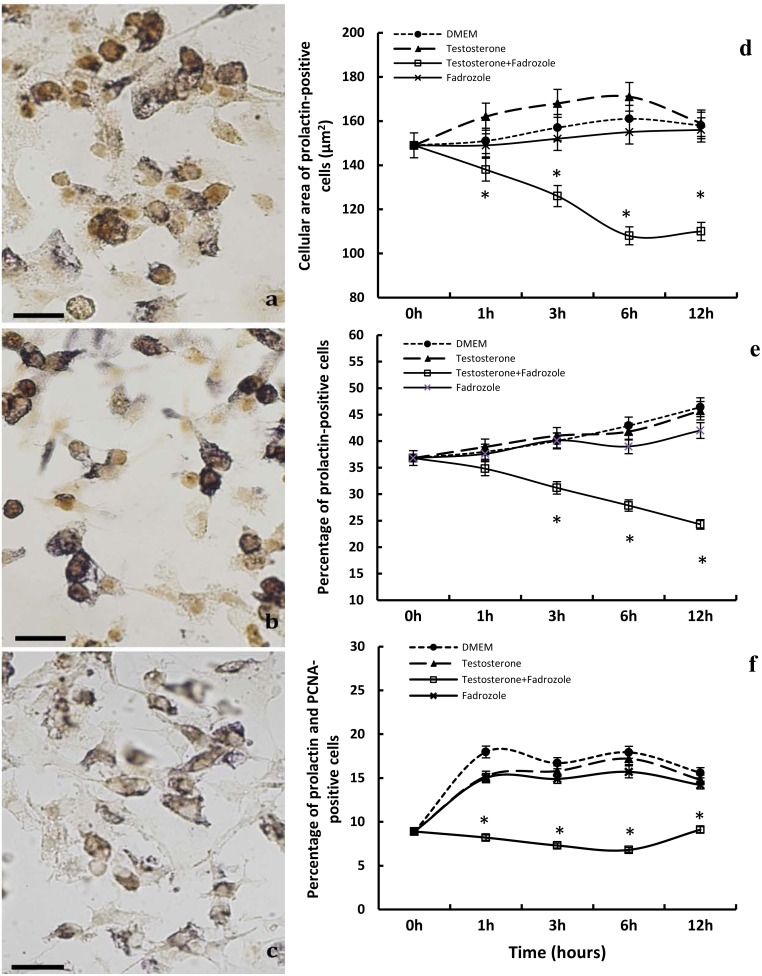
Effects of *in vitro* treatment with fadrozole on prolactin-positive cells. Double immunocytochemical staining for PCNA (brown) and prolactin (dark blue-grey) in control dishes a), testosterone-treated dishes b), and testosterone- and fadrozole-treated dishes c). Scale bar: a,b,c: 50 µm. d) Plot showing the decrease in cellular area at the different time-points assayed; from 3 to 12 hours of treatment with testosterone and fadrozole a significant decrease (*p<0.01) was observed. e) The percentage of prolactin-positive cells decreases as from 3 to 12 hours of treatment with testosterone and fadrozole (*p<0.01). f) The percentage of PCNA- and prolactin-positive cells, out of the total number of prolactin-positive cells, decreases significantly from 1 to 12 hours of treatment with testosterone and fadrozole (*p<0.01).

No statistically significant variations in the cellular area of prolactin-positive cells were found following treatment with fadrozole or testosterone alone (Figure 4d). Treatment with fadrozole plus testosterone significantly decreased the size of the prolactin-positive cells with respect to the other treatment groups analyzed. This effect was statistically significant from 3 to 12 hours of treatment (p<0.01).

#### Percentage of prolactin-positive cells

The variations observed among the control cultures (Figure 4a) and those treated with fadrozole or testosterone alone (Figure 4b) did not reach statistical significance (Figure 4e). In contrast, treatment with fadrozole plus testosterone (Figure 4c) significantly decreased the percentage of such cells as from 3 hours (p<0.01. Figure 4e).

#### Percentage of prolactin-proliferating cells

No significant differences were observed between the control cultures and those treated with fadrozole or testosterone alone (Figure 4f). The treatment with fadrozole plus testosterone (Figure 4c) significantly decreased the percentage of proliferating prolactin-positive cells as from the first hour of treatment (p<0.01. Figure 4f).

## Discussion

Previous findings from our laboratory suggest an important role of aromatase in the regulation of pituitary hormones, because we observed that aromatase P450 is expressed in the rat pituitary gland; that this expression is modified by sex and age, and that it could be involved in the genesis of spontaneous prolactinomas in old rats [3] or humans [4]–[12]. Moreover, aromatase mRNA has been reported in pituitary cells using *in situ* hybridization in trout [5]. These findings have been confirmed by other laboratories, in the sense that aromatase is expressed in the pituitary gland of different animal species, including humans [6], [10], [22], and that variations in its expression are regulated by gonadal steroids [6] because testosterone increased the expression but estradiol decreased it.

However, the role of aromatase in the regulation of pituitary hormones or cells is not well established, although several lines of evidence suggest the possibility that estrogens generated locally by aromatization could be involved in the activation of anterior pituitary mitotic activity [23]. Although androgens have an inhibitory effect on prolactin, the percentage of prolactin-producing cells in the pituitary glands of adult male rats is high [24]. Because the expression of pituitary aromatase is higher in males than in females [25], the local aromatization of testosterone to estradiol in the pituitary of males could be involved in the regulation of the population of prolactin-producing cells. The findings observed in the present study seem to support such a notion.

Androgenic effects on prolactin cells and secretion of the hormone are different, depending on whether the androgen is susceptible to its aromatization. Non-aromatizable androgens are inhibitors or hardly exert effects on prolactin producing pituitary cells, while aromatizable androgens increase the release of prolactin [26] and the levels of prolactin mRNA [27]. These functional changes obtained with doses of testosterone similar to those used in our study (10^−6^ to 10^−7^ M) are not accompanied by changes in cell growth [26]. These findings are consistent with those observed in our study, in which testosterone did not significantly modify the size or proliferation of prolactin-positive cells.

The in vitro inhibitory effects observed following treatment with fadrozole and testosterone jointly could be explained in terms of the androgenic effects of testosterone or because testosterone could be metabolized into non-aromatizable androgens.

Fadrozole is a potent non-steroidal inhibitor of aromatase P450, and it has been shown that a dose of 4 mg/Kg induces uterine atrophy without inducing adrenal hypertrophy [28]. In our in vivo study, we used a lower dose, within the ranges tested by other authors [29], [30] in a similar protocol to that described in [31], although slightly higher than the one used by these authors, with the aim of obtaining security inhibition rates higher than 90% [28], but not to induce atrophy of the organs of the reproductive system.

Estradiol is an important regulator of pituitary prolactin. It is well documented in the literature that estradiol, through coupling of the estrogen-estrogen receptor complex to the specific response element to estrogens in the promoter of the gene encoding prolactin synthesis, induces increases in prolactin synthesis through a biphasic transcription that culminates in an increase in the prolactin mRNA; this has a direct action on the gene able to alter the transcription [32]–[43]. Moreover, estradiol stimulates the secretion of prolactin [14], [19], [44]–[46].

These effects are accompanied by morphological changes such as hypertrophy of the cellular organelles [47]–[51], with evidence of hyperactivity and hyperplasia of prolactin-producing cells [34], [52], [53]. Furthermore, estradiol induces the proliferation of pituitary prolactin-producing cells [20], [54]–[58].

In the present study, after *in vivo* treatment with the aromatase antagonist fadrozole, highly significant changes were observed in pituitary prolactin-positive cells: decreases in the intensity of the immunohistochemical cytoplasmic reaction, decreases in the percentage and proliferation of prolactin-positive cells, and decreases in the cellular and nuclear areas, accompanied by lower levels of serum prolactin. All these findings suggest a lower activity in prolactin cells of fadrozole-treated animals than in control or untreated animals. Similar findings were observed when the effect of fadrozole on prolactin-producing cellswas compared *in vitro* after testosterone treatment.

Differences in the intensity of the cytoplasmic reaction could be explained in terms of decreases in the intracellular amount of prolactin, as has been previously documented at our laboratory for pituitary prolactin-producing cells, and this is supported by the decreases in serum prolactin levels found in the present study.

Decreases in cellular and nuclear areas support the notion that the cellular activity and/or cellular proliferation of prolactin cells and hormonal secretion is decreased, as demonstrated in previous studies [59].

As may be deduced from the results of this study, and in accordance with previous studies, aromatase is clearly expressed in the pituitary of male rats, and pituitary aromatase is probably expressed in different endocrine cells, including prolactin-producing cells [6]. This observation is very relevant because in the male pituitary aromatizable androgens can be metabolized to estrogens by means of aromatase; thus, estrogens can modulate and regulate the population of prolactin-producing cells, accounting for the important percentages of these cells in the male pituitary [24].

Because estradiol induces effects opposite those of fadrozole on prolactin cells, and since the aromatization of testosterone to estradiol is mediated by aromatase, the effects of fadrozole observed in our study suggest that aromatase would be involved physiologically in the regulation of the pituitary prolactin-producing cells of male rats, transforming testosterone to estradiol at pituitary level.
